# The costs of HIV prevention for different target populations in Mumbai, Thane and Bangalore

**DOI:** 10.1186/1471-2458-11-S6-S7

**Published:** 2011-12-29

**Authors:** Sudha Chandrashekar, Anna Vassall, Bhaskar Reddy, Govindraj Shetty, Peter Vickerman, Michel Alary

**Affiliations:** 1 London School of Hygiene and Tropical Medicine, London, UK; 2 St Johns Research Institute, India; 3 Karnataka Health Promotion Trust, Bangalore, India; 4 Centre hospitalier affilié universitaire de Québec, Canada

## Abstract

**Background:**

Avahan, the India AIDS Initiative, delivers HIV prevention services to high-risk populations at scale. Although the broad costs of such HIV interventions are known, to-date there has been little data available on the comparative costs of reaching different target groups, including female sex workers (FSWs), replace with ‘high risk men who have sex with men (HR-MSM) and trans-genders.

**Methods:**

Costs are estimated for the first three years of Avahan scale up differentiated by typology of female sex workers (brothel, street, home, lodge based, bar based), HR-MSM and transgenders in urban districts in India: Mumbai and Thane in Maharashtra and Bangalore in Karnataka. Financial and economic costs were collected prospectively from a provider perspective. Outputs were measured using data collected by the Avahan programme. Costs are presented in US$2008.

**Results:**

Costs were found to vary substantially by target group. Non-governmental organisations (NGOs) working with transgender populations had a higher mean cost (US $116) per person reached compared to those dealing primarily with FSWs (US $75-96) and MSWs (US $90) by the end of year three of the programme in Mumbai. The mean cost of delivering the intervention to HR-MSMs (US $42) was higher than delivering it to FSWs (US $37) in Bangalore. The package of services delivered to each target group was similar, and our results suggest that cost variation is related to the target population size, the intensity of the programme (in terms of number of contacts made per year) and a number of specific issues related to each target group.

**Conclusions:**

Based on our data policy makers and program managers need to consider the ease of accessing high risk population when planning and budgeting for HIV prevention services for these populations and avoid funding programmes on the basis of target population size alone.

## Background

It is estimated that around 2.5 million people were living with HIV/AIDS in India in 2006 [1-3]. Much of the HIV transmission in India occurs within networks of individuals who have high levels of risk [4]. The India AIDS Initiative or Avahan Programme is a large scale 10-year HIV-prevention programme in the six Indian states most affected by the HIV epidemic - complementing programs by the Government of India. In its first five years, Avahan focused on core and bridging populations in order to reduce the spread of HIV in these populations as well as in the general population [5].

Avahan delivers HIV prevention services to a wide range of high risk populations. Female sex workers (FSW) are the largest group, with Avahan targeting an estimated 310,000 FSWs. In India, FSWs are categorized into different typologies, based on where they recruit or solicit clients and not where they live or entertain the clients [6,7]. The major typologies are street based (SB), bar based (BG), brothel based (BB), lodge based (LB), home based (HB), dhaba based and highway based. These categories are often overlapping and fluid [4]. Avahan mapping in four southern states found that 60% of female sex work in India is street based, 9% brothel based, 12% are lodge based, 19% home based and others. Avahan also targets around 123,000 high risk “men who have sex with men” (HR-MSMs) and transgenders. Transgenders include hijras. While one sub-set of hijras is involved in blessing during births, marriages and ceremonies, another is involved in begging, and a third is involved in sex work.

There are a limited number of peer reviewed studies on the costs of HIV prevention services in Asia [8-14]. These show that costs vary considerably by setting, finding that the cost of reaching a sex worker ranges from US$10 to US$124 (US$2006) [14-17]. There are many reasons for these differences, foremost of which is scale [15]. However, other factors such as the type of the population reached, programme intensity, age of the programme may also impact costs. For example, a study by Dandona et al in 2008 found that costs of similar HIV prevention interventions fell as scale increased and over time [17].

As a part of the overall evaluation plan, Avahan was subject to an intensive costing effort and an economic evaluation in four southern states, Karnataka, Maharashtra, Andhra Pradesh and Tamil Nadu during 2005-2008. This evaluation covers over eighty districts, and thus provides an opportunity to understand the drivers of HIV prevention costs [18]. This paper presents the costs of delivering Avahan HIV prevention package in two urban settings where distinct typologies of high risk population were targeted in order to explore how the costs vary for different typologies in similar settings.

## Methods

### Study setting

This study presents the costs of HIV prevention in two large scale urban settings, Mumbai and Thane in Maharashtra and Bangalore in Karnataka. In Mumbai and Thane, we measured the costs of delivering HIV prevention to different typologies of FSWs, HR-MSM and transgenders. A cross sectional behavioural and biological survey conducted in April 2006 in Maharashtra found an HIV prevalence of 28.1% (22.2-34.8) among brothel-based FSWs and 19.2%(13.7-26.2) among street-based FSWs [19]. In Bangalore, we measured the costs of HIV prevention services to FSWs and HR-MSMs. A study of HR-MSM in Bangalore in-2008 found an HIV prevalence of 18.9% [19]. HIV prevalence among the FSW population in Bangalore was estimated to be 12.6% from routine surveillance [20].

### Programme description

In Mumbai/Thane, Avahan funds 16 separate non-governmental organizations (NGOs) to deliver HIV prevention services. Each of these NGOs targets different high risk populations (Table 1). The estimated population targeted by Avahan in Mumbai and Thane was 34,919 persons. By the end of year three, a total of 51,885 individuals had been reached at least once. The number of individuals reached was higher than the population estimate due to the migration of individuals in and out of the target group. The breakdown of the population reached by Avahan in Mumbai and Thane consisted of: 51% bar- based FSWs, 13% brothel -based FSWs, 16% home -based FSWs, 12% street- based FSWs, 4% HR-MSMs and 3% transgenders. Programme interventions included community mobilisation, advocacy, crisis management, outreach, behavioural change and communication (including innovative strategies to reach out to the key population), sexually transmitted infections (STI) services, counselling and condom promotion and provision. These services are considered an essential package of services and are delivered for all target groups [21]. NGOs, however, are allowed to decide the intensity of (frequency of contact with the programme staff) and the way in which interventions are delivered.

In Bangalore, HIV prevention is implemented by two community based organizations, one targeting street-based FSW and the other HR-MSM. The estimated target population was 18,969 persons, and by year three, 36620 people had been reached at least once a year (Table 1). The intervention package was similar to that in Mumbai and Thane and comprised of outreach activities including peer led behaviour change communication, STI services and condom promotion [22]. Community mobilisation included drop-in centre activities, special events; welfare activities for the key population and enabling environment activities include advocacy, sensitization of stakeholders and crisis management.

**Table 1:** NGO site characteristics, estimated population, and population reached

NGO/CBO	Typology*	Estimated population **	Total population reached by year 3
1	BB	2000	3381
2	BG	2200	2772
3	BB / BG	1700	2194
4	HB / BG	3000	4384
5	HR-MSM	1500	2411
6	BB / LB / SB / HB / BG	1500	1659
7	HIJRA	1500	1125
8	BB / HB/ BG	4057	5251
9	HB / BG /MSW	2800	3676
10	BB / BG	2671	3451
11	BB / SB / HB	1800	2751
12	BG / MSW	3052	5201
13	BB / LB / SB / HB / BG	2039	3646
14	HIJRA	1800	1668
15	BG / SB	2000	3482
16	BB / LB / SB / HB / BG	1300	4833

	**Total Maharashtra**	**34919**	**51885**

**Bangalore, Karnataka**			

17	SB	12743	25124
18	HR-MSM	6226	11496

	**Total Karnataka**	**18969**	**36620**

* Street-based (SB), brothel-based (BB), lodge-based (LB), home-based (HB), bar girls (BG) and high risk men who have sex with men (HR-MSM).

** Estimated using community mapping surveys.

### Costing methods

Our methodology is based on the UNAIDS Costing Guidelines for HIV Prevention Strategies [23] as recommended by Asian development bank. An ingredients-based costing methodology was used to consider both financial and economic costs from the provider perspective - including both implementation and support costs. The ingredients approach identifies the inputs required to deliver the intervention, and then measures and values them.

Five NGO sites were chosen for extensive field work, representing the range of typology of sex work interventions implemented, (brothel based FSWs; street based FSWs, HR-MSM, bar girls and transgenders). For each NGO, data was collected on project activities, financial expenditure and outputs. Time-sheets were used to determine allocation of resources between different activities and population groups. For all the other NGOs studied, data was collected from their routine reports and no fieldwork was done. Expenditure data was obtained from routine financial and management reporting, staff records and interviews with staff. We estimate both financial and economic costs. Financial costs represent the money spent by the programme to deliver the intervention, whereas economic cost includes the value of all inputs (including the value of resources that may be donated). Therefore, for the detailed costing sites, data on donated goods and services were also collected from the programme. The economic costs of these items were valued at market prices obtained from local shops and interviews with project staff.

Costs were classified according to three characteristics: the phase of implementation, organisational level where costs are incurred and type of cost. The time period between the decision to implement an intervention and starting its delivery to the beneficiaries was defined as the start up phase. All costs incurred in the start-up period were annualised to reflect utility beyond the start up period. All costs incurred after the start up period were defined as implementation costs. Costs were collected both from the state level (supporting the NGOs) and from each NGO. Costs are categorised as either recurrent or capital costs using a definition of capital cost to be an item with a useful life of more than one year. Capital costs include equipment, furniture and fixtures, vehicles, rental deposits and start up costs. Capital equipment was assumed to have a life of between 5 and 10 years, depending on the item. Capital costs were annualised to reflect the utility of their use during the course of the programme. A discount rate of *3%* was used. Recurrent costs include all personnel costs, travel, building operating and maintenance supplies, cost of condoms, medical supplies and all other supplies costs.

At the start of an intervention in a district or sub-district, NGOs conducted a formal external mapping and size estimation exercise. Some state-level lead implementing partners updated these numbers on a regular basis (every 12 to 18 months) using programme data- others conducted formal size estimation exercises; others used programme data [24]. Programme output data was sourced from the programme Management Information System (MIS) which captures the number of individuals reached, those contacted monthly by outreach workers and number of individuals attending STI services [25]. Programmes were designed to cover high risk individuals in specific geographic area and as such did not follow the individuals when they left the area. All data were entered into a specifically designed MS Excel workbook.

Since the cost estimates cover more than one year of expenditure, where relevant, costs have been adjusted to US$2008 using the Gross Domestic Product (GDP) deflator reported by the Indian Ministry of Finance [26]. Further details on the cost analysis methods are explained in Chandrashekar et al (2010) [15].

## Results

The estimated target population, population reached, contacts made and clinic visits per year by typology of high-risk population are presented in Table 2. An examination of the number of contacts made per person reached shows that programme intensity differed by target population typology. For example, in Mumbai/Thane on average, by 2008, each brothel- based and bar-based FSW reached was contacted around 4.4-4.8 times per year. Street- based FSWs were contacted less frequently at around 4.2 times per year. In comparison, while intensity was low to begin with, by 2008 home based sex workers were contacted 5.4 times a year. Similarly HR-MSM intensity was low initially, but by 2008 was 5.9 times per year. In Bangalore, the number of contacts per year was found to be about 4.4 times a year for FSWs and 4.9 times a year for HR-MSMs in 2008. By 2008, in Mumbai/ Thane, STI clinic visits a year did not vary substantially by population group and the mean was around 1 per year. For Bangalore, the mean frequency of clinic visits per year for HR MSMs was around 0.47 in 2008. For FSWs it was slightly higher at 0.58 clinic visits per year.

**Table 2:** Programme outputs for Mumbai/Thane and Bangalore, 2005-8

Typology	Population estimation			Population reached at least once a year			Contacts per year, per person reached			Clinic visits per year, per person reached		
**Year**	05-06	06-07	07-08	05-06	06-07	07-08	05-06	06-07	07-08	05-06	06-07	07-08

Brothel-based	3679	4044	4112	3489	4958	6818	8.1	4.9	4.8	0.5	1.0	1.0
Lodge-based	344	369	254	236	606	573	3.1	4.1	4.1	0.7	0.8	0.9
Street-based	3376	3754	3619	3356	4906	6622	7.9	5.1	4.2	0.5	1.0	1.0
Home-based	3079	4909	6027	2497	5670	8328	2.2	4.6	5.4	0.9	1.1	1.1
Bar girls	14373	16900	16092	12937	20060	24317	3.2	3.9	4.4	0.5	0.9	1.0
HR-MSM	1502	1503	1515	2314	2324	2434	3.1	6.4	5.9	0.1	0.9	1.1
Hijras*	1635	3440	1800	1284	2875	1668	16.1	6.6	3.8	0.5	0.7	0.8

**Total Mumbai/ Thane**	**27990**	**34919**	**33419**	**26,113**	**41399**	**50760**	**5.0**	**4.6**	**4.7**	**0.5**	**0.9**	**1.0**

**Bangalore, Karnataka**												

HR-MSM	6226	6226	6226	3591	9483	11496	3.7	4.1	4.9	0.3	0.3	0.5
FSW	12929	12743	12743	9241	22476	25124	1.7	4.0	4.4	0.5	0.4	0.6

**Total Bangalore**	**19155**	**18969**	**18969**	**12832**	**31959**	**36620**	**2.2**	**4.0**	**4.6**	**0.5**	**0.4**	**0.5**

*one NGO closed

Female sex workers (FSW) and high-risk men who have sex with men (HR-MSM).

Table 3 presents total programme costs broken down by typology and risk group for all sites in Mumbai, Thane and Bangalore. Broadly, total costs of each programme increased over the years, with the most the increase occurring between years 1 and 2. The total economic cost is US$9.2 million over the three years in Mumbai/Thane. 44% of this cost was spent on interventions focussed on bar based sex workers. The allocation to different target groups (as a proportion of total cost) remained much the same throughout the period, (aside from the cost of reaching home based workers that increased as the programme expanded).The total cost of the programme in Bangalore was around US$3.1 million. The proportion cost related to HR-MSM, increased over time and reached around 34.4% of the total cost by 2008.

**Table 3:** Programme implementation costs by Typology and high risk group (US $ 2008) (Economic 3%)

Typology		Costs by typology (discount rate 3%)						
**Year**	**05-06**	%	**06-07**	%	**07-08**	%	**Total**	%

Brothel based	368936	15.1	435025	13.1	500151	14.3	1304112	14.1
Lodge based	32818	1.3	42390	1.3	32327	0.9	107535	1.2
Street based	318096	13.0	417830	12.6	440427	12.6	1176353	12.7
Home based	259848	10.6	445463	13.4	624896	17.8	1330207	14.4
Bar girls	1135975	46.5	1452101	43.7	1516250	43.3	4104326	44.3
HR-MSM	164067	6.7	194223	5.8	190603	5.4	548893	5.9
Hijras	162968	6.7	333946	10.1	196651	5.6	693565	7.5

**Total**	**2442708**	**100.0**	**3320978**	**100.0**	**3501305**	**100.0**	**9264991**	**100.0**

FSW site Bangalore	446228	73.6	739482	65.2	922722	65.6	2108432	67.0
HR-MSM site Bangalore	160181	26.4	394701	34.8	484476	34.4	1039358	33.0

**Total**	**606409**	**100.0**	**1134183**	**100.0**	**1407198**	**100.0**	**3147790**	**100.0**

FSW – female sex worker, HR-MSM – high-risk men who have sex with men.

**Figure 1:**
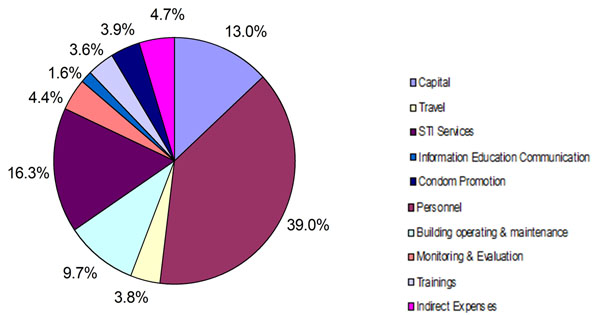
Cost by input type for all sites in Mumbai/Thane and Bangalore, 2005-2008 (%).

**Figure 2:**
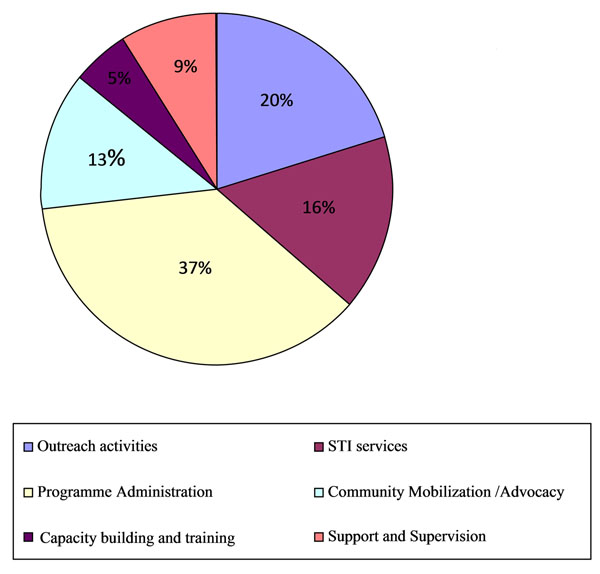
Cost by activity for all NGO sites in Mumbai/Thane and Bangalore, 2005-2008 (%).

Figure 1 shows cost breakdown by input type across all areas studied. Capital costs account for around 13% of total cost. Personnel costs account for 39% of cost, followed by STI supplies costs at 16%. Figure 2 shows the breakdown in terms of activities. Programme administration costs (including mapping, programme monitoring and management information system, start-up activities, management staff, office expenses and overheads) account for around 37% of total costs, followed by outreach at 20%, STI services at 16% and, community mobilisation and advocacy activities at 13 %. Start up activities contributed around 5% of the total costs.

**Figure 3:**
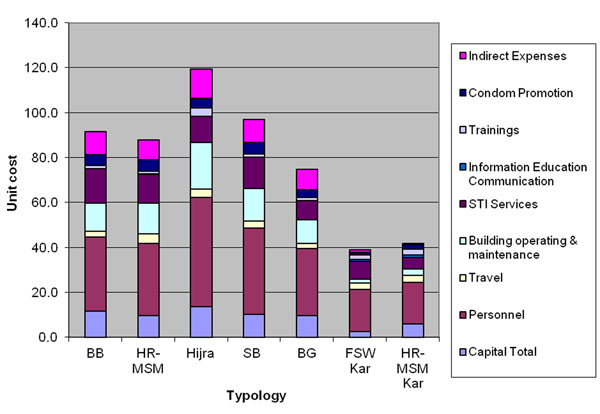
Economic unit costs of detailed NGO costing sites in Mumbai/Thane and Bangalore by input and typology US$2008 (3% discount rate).

**Figure 4:**
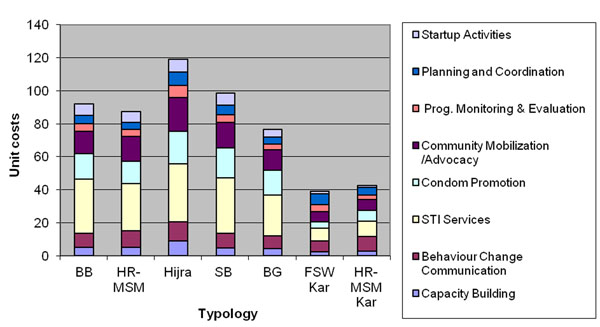
Economic unit costs of detailed NGO costing sites in Mumbai/Thane and Bangalore by activity and typology (detailed costing sites only) US$2008 (3% discount rate).

The unit costs by typology (estimates made from detailed costing sites/ or single target population sites only) are shown in Table 4. By year 3, the unit costs of reaching a member of the targeted population ranged from $US 75 to $US 116 in Mumbai and Thane and $US 37 to US$ 42 in Bangalore. Costs for non-fixed location groups (Hijras, street- based FSWs and HR-MSMs) tended to be higher than those for operating from a fixed setting (brothel and bar- based FSWs). Table 4 also shows that costs per person reached decline over time for brothel and bar based workers in Mumbai and Thane and in all target populations in Bangalore, but not for other groups. Figure 3 shows the cost breakdown for each typology by input type. For the most part, harder to reach groups (e.g. the non-fixed location groups) have higher capital (including building maintenance) and personal (including training) costs per person reached than groups operating from fixed settings. In terms of breakdowns by activity, harder to reach typologies show a similar proportional breakdown of inputs as fixed location groups (Figure 4). We also analysed the relationship between the numbers of contacts per person reached per year (from Table 2) and the unit cost per person reached (Table 4). Some patterns were observed. For example in year 2 intensity and cost follow the same trends. However both in year 1 and 3 this association is weaker. Overall we found a correlation co-efficient(r=0.32), but this was not found to be statistically significant.

**Table 4:** Unit costs of detailed NGOs by Typology (Economic costs 3%)

Typology	Population estimation			Cost per population reached			Cost per contact		
**Year**	**05-06**	**06-07**	**07-08**	**05-06**	**06-07**	**07-08**	**05-06**	**06-07**	**07-08**

Brothel-based	104	107	121	103	97	75	10	21	17
Street-based	100	123	124	93	102	96	7	15	18
Bar girls	98	86	97	92	86	75	25	18	10
HR-MSM	123	145	145	80	94	90	26	15	15
Hijras	-	101	107	-	123	116	-	43	30

HR-MSM site in Karnataka	26	63	78	45	42	42	12	10	9
FSW site in Karnataka	35	58	72	48	33	37	28	8	8

## Discussion

There is considerable interest by the Government of India, and other countries to sustain HIV prevention programmes, but their cost remains a concern. Improving the knowledge of factors that drive costs can assist programme budgeting and help in deciding the optimal resource allocation required to deliver these programmes. Previous resource estimates have tended to deal with financial costs only and assumed that they were linear across population typologies [26]. Many governments and programme also determine their budgets by allocating a standard cost for reaching a member of a target group, and multiplying this by target population size. Evidence to date has primarily focussed on establishing the extent of the relationship between costs and the scale and timing of programme implementation, and the characteristics of the (recipient) target group.

We find that the costs of reaching different populations with HIV prevention interventions in the similar settings vary substantially. The costs for all typologies are at the higher end of those found in previous studies in India of both Avahan and HIV prevention delivered by others [11,14-17]. This is likely to be due to the package of services included and the fact that our costs also include expenditures beyond the NGO level, which most previous studies omit. However, broadly our findings suggest that sex worker populations who operate from non-fixed locations are likely to cost more. For example, in Mumbai/ Thane, the cost of reaching hijras is approximately 1.5 times the cost of reaching the lowest cost group (bar girls). In Bangalore, we find that the cost of reaching HR-MSMs is with marginally higher than that of reaching FSWs. This is somewhat different than the findings of the one previous study from India that reports on the costs of reaching these two groups in the same setting [11]. This found that the mean costs of the HR-MSM programme and reaching FSWs in Andhra Pradesh were, respectively, US$7.8 and US$32.1 (US$2006) per person reached. The reasons for this difference are hard to ascertain without a detailed understanding of what was included in the HR-MSM cost in that study.

The factors that drive cost differences between high-risk population groups are complex. As with between settings, unit cost variation within similar settings is likely to be related to the size of the target group. This is demonstrated by the decreases in unit costs over time as the programme expanded (Table 4). Moreover, in any one year, larger populations groups, such as bar girls, have lower costs (Table 4). In the few instances where this pattern cannot be observed, the explanation was found to be due to site-specific issues. For example, NGOs changed condom supplier over the years, and this increased costs over time. This scale effect may also explain the difference in costs between reaching FSWs and HR-MSMs in Bangalore, given that the FSW population is more than twice the size of the MSM population. These finding mirrors those from earlier studies by Dandona et al (2008) [17] and Chandrashekar et al (2010) [17].

The scale effect within NGO sites for different target groups is also illustrated by our cost breakdowns. Our cost breakdowns show that the proportion of both capital and personnel related costs are substantially higher per person reached in smaller target groups whereas other costs such as those for STI supplies (as a proportion of unit costs) remain more uniform across different target groups . This is likely to be due, in part, to the fact that each NGO needs a certain level of fixed capacity in key areas, such as support and supervision for outreach workers. Planners and funders therefore need to consider whether it is worth encouraging NGOs targeting smaller groups to share these fixed costs between one another, and explore how their funding mechanisms can better encourage the more efficient use of fixed resources.

Our data also suggest that higher intensity of service, in terms of numbers of contacts made, is associated with higher unit cost per person reached, albeit in a very limited way. In Mumbai/ Thane the magnitude of the difference in intensity (ranging from 4.2 to 6.6 contacts per year) is aligned to magnitude of the cost differences observed, with the exception of street–based FSWs, particularly in year 2. In year three there were specific issues with the management of the Hirja programme that meant that costs remained high, despite a lower intensity of effort. In Bangalore however, this is much less the case, as differences in intensity are much lower, and thus overall we found no statistically significant relationship between intensity and cost. More work needs to be done with large sample sizes to explore this relationship further.

We observe little variation in the proportion of activity costs between different population groups. This indicates that no group required a special mix of activities, but nevertheless, when asked to interpret our findings programme staff identified some specific issues when working with the non-fixed location groups, such as hijras. For example, it took longer for staff to orientate themselves regarding the nature of the hijra population. Moreover, it took time to build rapport with the hijra population; requiring a higher frequency of visits from the NGO staff compared than other groups. Programme staff also highlighted issues with keeping track of street -based FSWs repeatedly, due to the highly mobile nature of that population. In addition, street-based FSWs were considered more reluctant to participate in the project services, because of the time commitment and the corresponding loss of clients and income. Street- based FSWs were also worried that visiting drop-in centres would reveal their identity and lead to more stigma and harassment from police and the local community. The factors were thought by the NGOs to increase the level of outreach and thus cost of reaching these groups.

This study is limited by various factors, the most important of which is its small sample size. Ideally when exploring cost differences between different groups one would use statistical techniques, examining costs and cost drivers over a large number of sites. However, even in such large scale settings, it is difficult to capture the detailed cost data required from a sufficient number of NGOs to enable this analysis. We are planning a follow-up econometric of costs drivers’ analysis of all Avahan sites, but as few sites target specific high risk sub-populations, there are an insufficient number of sites to explore the full impact of typology on cost statistically. We therefore needed to rely on the descriptive analysis presented above. An alternative method would be to measure costs at the client/ individual population level, and it is recommended that future studies explore opportunities to do this.

Furthermore, our costs are also likely to be impacted by a number of other factors beyond target population. While the settings were similar in terms of NGO characteristics and HIV prevalence, other factors related to implementation are likely to also have an impact on costs. For example the programme had some difficult phases in particular districts in Mumbai due to a major bomb blast in the metro and combing operations by the police. There was sudden closure of bars due to government instruction and the programme had to change the strategy to reach the sex workers in the place of residence. The issue of frequent raids in brothels also affected programme services in certain areas. Again this limits the robustness of results, even though most of the above effects were temporary in nature.

Our findings suggest that policy makers, planners and analysts should consider the typology of the target population when conducting efficiency analyses and setting budgets across HIV prevention programmes. Analytically, care should be taken to judge costs and efficiency in the context of the populations they service. However, setting budgets using a fixed amount per person reached risks penalising those NGOs who are targeting more difficult to reach groups and may create a perverse incentive to focus on high risk groups that cost less to reach.

## Conclusion

Different HIV prevention target groups present multiple issues in delivery of services and interventions, reflected in the cost variation. Policy makers and programme managers are therefore recommended to examine the particular circumstances of the populations being reached when setting budgetary limits for HIV prevention services for high risk groups.

## Competing interests

The authors declare that they have no competing interests.

## Authors' contributions

SC: contributed to the design, data collection, analysis, interpretation and prepared the first draft of the paper; AV: contributed to the analysis, interpretation of the data and the manuscript; PV: contributed to manuscript preparation; BR and GY: assisted in the data collection, data entry, preliminary data analysis and generation of tables; MA: Principal Investigator of the main study and contributed to the design of the study.

## Source of funding

This research was funded by the Bill & Melinda Gates Foundation and also financial support from HIV Research Trust UK.
